# Interactions Among Host–Parasite MicroRNAs During *Nosema ceranae* Proliferation in *Apis mellifera*

**DOI:** 10.3389/fmicb.2018.00698

**Published:** 2018-04-10

**Authors:** Jay D. Evans, Qiang Huang

**Affiliations:** ^1^Bee Research Laboratory, United States Department of Agriculture-Agricultural Research Service (USDA-ARS), Beltsville, MD, United States; ^2^Institute of Bee Health, Vetsuisse Faculty, University of Bern, Bern, Switzerland

**Keywords:** honey bee, *Nosema ceranae*, microsporidia, RNAi, miRNA

## Abstract

We previously identified microRNA (miRNA) from *Nosema ceranae* and found that knockdowns of transcripts for the parasite protein *Dicer* greatly reduce parasite reproduction. In order to study parasitic miRNA functions and identify candidate target genes, we fed honey bees infected with *N. ceranae* with small interfering RNA (siRNA) targeting the *N. ceranae* gene *Dicer*. We then deep-sequenced honey bee and *N. ceranae* miRNAs daily across a full 6-day proliferation cycle. We found seven honey bee and five *N. ceranae* miRNAs that were significantly differently expressed between the infection and siRNA-Dicer groups. *N. ceranae* miRNA showed potentially strong impacts on the *N. ceranae* transcriptome, where over 79% of the total protein coding genes were significantly correlated with one or more miRNAs. *N. ceranae* miRNAs also can regulate honey bee metabolism and immune response, given parasitic miRNAs were secreted into the cytoplasm. Our results suggest that *N. ceranae* miRNAs regulate both parasite and host gene expression, providing new insights for microsporidia parasitism evolution.

## Introduction

MicroRNAs (miRNAs) are a group of small regulatory RNAs, which regulate gene expression at the post-transcription level. MiRNAs are usually 22 nucleotides long with a seed region (2–8 bp) that binds to the 3′ untranslated region (UTR) of a mRNA, generally leading to degradation (Bartel, 2004). miRNAs have diverse expression patterns and regulate various developmental and physiological processes (He and Hannon, 2004). Furthermore, miRNAs can act as an infection mechanism adopted by parasites to modulate their host immune responses (Weiberg et al., 2013; Buck et al., 2014).

*Nosema ceranae* is a unicellular fungal parasite which infects honey bee mid-gut epithelial cells (Fries et al., 1996). The intracellular proliferation cycle of *N. ceranae* lasts approximately 4 days and the parasite starts to reproduce offspring spores soon after entering the mid-gut lumen (Higes et al., 2007; Gisder et al., 2011). Infected cells eventually release a large number of spores, which could again germinate in the lumen and infect neighboring epithelial cells. Microsporidian parasites have compact genomes and most parasite species have lost the RNAi pathway (Ndikumana et al., 2017). However, *N. ceranae* maintains three key RNAi gene orthologs, *Dicer*, *Argonaute* and *RNA-dependent RNA polymerase*. Suppressing the gene expression of *Dicer* significantly reduces *N. ceranae* spore production (Huang et al., 2016). To date, *N. ceranae* is the only microsporidian species with identified miRNAs (Huang and Evans, 2016). The functions and target genes of those parasitic miRNAs remain unclear. To fill this gap, we suppressed the gene expression of *Dicer* with small interfering RNA (siRNA) and deep sequenced the miRNAs of both host and parasite. We compared this study with a previous miRNA sequencing study (Huang et al., 2015) to determine whether siRNA-Dicer treatment can reverse host miRNA expression patterns in infected honey bees. The results clarify how *N. ceranae* interrupt honey bee defenses, aiding control strategies for other intra-cellular parasites.

## Materials and Methods

### Parasite Infection and Sampling

*Nosema ceranae* spores were isolated from the mid-guts of heavily infected honey bee workers from multiple colonies. The spores were purified using a Percoll gradient procedure and used to infect bees which were then sampled as described previously (Huang et al., 2016). For each of the four treatment groups, eighty newly emerged workers were fed individually with 2 μl of sucrose solution as follows: (1) without spores, as the uninfected group; (2) with 10^5^ *N. ceranae* spores without siRNA treatment, as the infection group; (3) with10^5^ *N. ceranae* spores and 1.5 μg siRNA (targeting the *Nosema* gene, *Dicer)* as the siRNA-Dicer group; and (4) with 10^5^ *N. ceranae* spores and 1.5 μg scrambled siRNA, as the siRNA-scramble group. The sequences of the designed siRNA were provided in a previous study and the scrambled siRNA did not match either the host honey bee or *N. ceranae* genomes (Huang et al., 2016). Each group of honey bees was split into two rearing cups of 40 bees each. For each day’s post-infection (dpi), bees were frozen and total RNA was extracted from mid-gut tissues from five honey bees individually per treatment using TRIzol from 1 to 6 dpi. In this study, equal amounts of RNA from these samples were pooled and small RNA were separated with a small-RNA Sample Prep Kit for ILLUMINA sequencing. Two sequencing libraries were prepared for each treatment at each of the six sampling points, totaling 36 sequencing libraries.

### Small RNA Sequencing Analysis

Raw sequencing reads have been deposited at NCBI bio-project PRJNA399493. Predicted hairpin and mature honey bee miRNA sequences were downloaded from miRBase (version21). The hairpin and mature miRNA sequences of *N. ceranae* were obtained from a previous publication (Huang and Evans, 2016). Small RNA reads were aligned to the *Apis mellifera* (Amel 4.5) and *N. ceranae* (ASM98816v1) genomes to extract the counts of mature miRNAs via the miRdeep2 package (Friedländer et al., 2012). The original counts were normalized with weighted trimmed mean of *M*-values (TMM) to calculate relative expression levels. Two replicates for each treatment were used to calculate common dispersion and the *P*-values for each of the protein genes among three groups using edgeR package (Robinson et al., 2010). The expression levels of each miRNA were pair-wise compared among the *N. ceranae* infection group, siRNA-Dicer group and siRNA-scramble group. To be considered significantly expressed, honey bee miRNAs needed to meet three criteria (1) differential expression between the infection group and the siRNA-Dicer group, (2) differential expression between the siRNA-Dicer group and siRNA-scramble group, and (3) no differences in expression between the siRNA-scramble group and non-siRNA control group.

Every raw-read count has substantial impact on the subsequent normalized read count because of the low numbers of parasite miRNAs and time-specific expression patterns (Huang and Evans, 2016). It is difficult to determine the differential expression of miRNAs using methods adopted for mRNAs. Instead, a time series analysis method was used to determine if siRNA-Dicer feeding significantly changed the expression pattern using the data from all 6 days post-infection using maSigpro package (Nueda et al., 2014).

The target genes of miRNAs were predicted using sequence-based algorithms. Annotated 3′UTRs of protein-coding genes were used for miRNA target prediction using the Miranda package (Enright et al., 2003). Next, target gene predictions were strengthened using an expression-based method. Co-expression between significantly regulated miRNAs and the transcriptome was analyzed with the WGCNA package across all 36 libraries (Langfelder and Horvath, 2008). Only candidate genes predicted by both methods were maintained for functional analysis. Fine-scale co-expression between miRNAs and mRNAs was performed again with WGCNA package. Transcriptome sequences were downloaded from NCBI bio-project PRJNA399493. The protein sequences of candidate genes were used to query the Pfam, UniProt, dcGO, and KEGG databases, to retrieve putative functions and involved pathways.

## Results

### Overall Sequence Reads Alignment Statistics

On average, over 13 million reads (19–35 bp per read) were generated from each small RNA library. The number of reads aligned to the honey bee genome decreased from 69.5% (infection group), 74.7% (scramble group), and 75.7% (siRNA-Dicer group) at 1 dpi (days post-infection) to 32.5% (infection group), 27.1% (scramble group), and 26.8% (siRNA-Dicer group) at 6 dpi. The post-infection day showed significant impact on the number of reads, which were aligned to the honey bee genome (ANOVA, *P* < 0.001). For the parasite, the number of reads aligned to the *N. ceranae* genome increased from 0.2% (infection group), 0.2% (scramble group), and 0.3% (siRNA-Dicer group) at 1 dpi to 32.1% (infection group), 42.3% (scramble group), and 43.7% (siRNA-Dicer group) at 6 dpi. The increase in alignments to parasite reads provides a measure of infection in the inoculated bees.

### Expression Profile of Honey Bee miRNAs and Candidate Target Gene Functions

**FIGURE 1:**
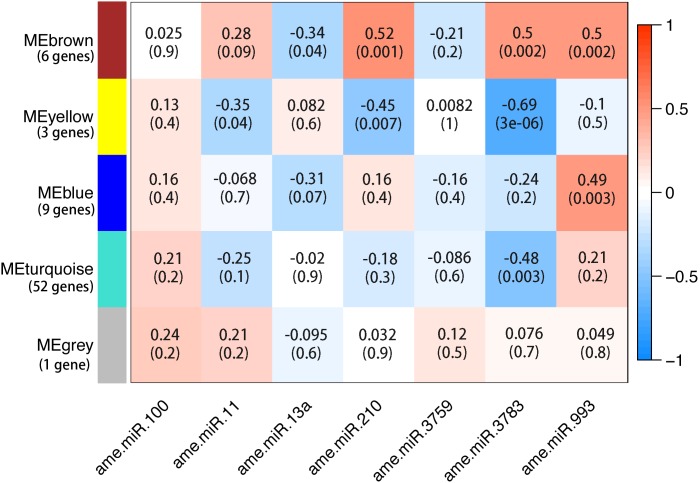
Association between honey bee microRNAs (miRNAs) and mRNAs at the expression level. The expression levels of miRNAs were treated as phenotype and the expression levels of mRNAs were treated as genotype. By correlating the genotype and phenotype, 71 mRNAs were clustered into five groups. The number of genes in each group was marked next to that group. Four of the five groups were significantly correlated with at least one miRNA. Furthermore, the 3′UTR of these 71 mRNAs match the seed region of miRNAs confirmed by sequence-based algorithm. If the target gene prediction is true, the miRNA should correlate with the target genes at the gene expression level. Each row corresponds to a gene cluster and each column corresponds to a miRNA. Each cell contains the corresponding correlation and *P*-value. The table is color coded by correlation to the color legend on the right side, which shows the range from lowest expression (blue) to highest expression (red). Data from all 36 libraries were included for the analysis.

Out of 259 described honey bee miRNAs, 178 miRNAs were detected and seven miRNAs were significantly regulated between the infection group and siRNA-Dicer group, after normalization with the siRNA-scramble group (**Table 1** and **Supplementary File S1**). Ame-miR-210 was significantly down-regulated at 3 dpi in the siRNA-Dicer group. Ame-miR-100 and ame-miR-11 were significantly down-regulated in the siRNA-Dicer group at 4 dpi. Ame-miR-3759 was significantly up-regulated in the siRNA-Dicer group at 4 dpi. Ame-miR-11, ame-miR-13a, and ame-miR-3783 were significantly down-regulated in the siRNA-Dicer group at 6 dpi. Ame-miR-993 was significantly up-regulated in the siRNA-Dicer group at 6 dpi. The distribution of up- and down-regulated miRNA was not significantly deviated from random (Chi-Square test, *P* > 0.05). Using sequence-based predictions, 204 mRNAs could be regulated by those seven significantly regulated miRNAs (**Supplementary File S2**). Out of these 204 mRNAs, expression of 71 mRNAs was significantly correlated with those seven miRNAs (**Figure 1**). By aligning the protein sequences to the KEGG pathway database, carbohydrate and lipid metabolism pathway modules, and nucleotide and amino-acid metabolism pathway modules were identified as being likely targets of these miRNAs, as well as components of the Wnt signaling pathway.

**Table 1:** Proportion of normalized reads from seven significantly differentially expressed microRNAs (miRNAs) between infection group and siRNA-Dicer group at each time points [*y* = count^infection^/(count^infection^+ count^siRNA-Dicer^)].

Host miRNAs	1 dpi	2 dpi	3 dpi	4 dpi	5 dpi	6 dpi
ame-miR-100	0.59	0.54	0.61	0.61^∗^	0.53	0.56
ame-miR-11	0.62	0.66	0.68	0.67^∗^	0.61	0.68^∗∗^
ame-miR-13a	0.51	0.43	0.43	0.45	0.59	0.61^∗^
ame-miR-210	0.51	0.56	0.64^∗^	0.55	0.55	0.67
ame-miR-3759	0.43	0.61	0.13	0.09^∗∗^	0.67	0.66
ame-miR-3783	0.52	0.54	0.51	0.54	0.56	0.61^∗^

*Values <0.5 indicate down-regulation in infection group compared with siRNA-Dicer group. Significant differential expression at ^∗^<0.05 and ^∗∗^<0.01, after normalizing siRNA-scramble group. dpi, days post-infection*.

### *N. ceranae* miRNAs in Two Genome Assemblies

Six miRNAs were identified based on a previous small RNA sequencing data set using the *N. ceranae* genome assembly (ASM18298v1) as a reference. In this study, the updated *N. ceranae* genome assembly (ASM98816v1) was used as a reference. By aligning the mature and hairpin sequences of miRNAs against the updated genome assembly, JL-1, JL-3, JL-4, and JL-5 were aligned to a unique position with 100% identity. The hairpin and mature sequences of JL-2 and JL-6 were aligned to multiple positions with gaps (**Table 2**).

**Table 2:** Comparing *Nosema ceranae* miRNAs in two genome assemblies. ASM18298V1 is the first *N. ceranae* assembly based on 454 sequencing.

Assembly		JL-1	JL-2	JL-3	JL-4	JL-5	JL-6
ASM18298V1	Position	Intergenic	Intergenic	Coding	Intergenic	Intergenic	Intergenic
	Identity	100%	100%	100%	100%	100%	100%
ASM98816V1	Position	Intergenic	Intergenic	Coding	Intergenic	Intergenic	Intergenic
	Identity	100%	94%	100%	100%	100%	94%

*ASM98816V1 is an updated N. ceranae genome assembly based on Illumina pair-end sequencing. JL-1, JL-3, JL-4, and JL-5 were aligned to both assemblies at unique positions. JL-2 and JL-6 were aligned to multiple positions and the position with best alignment identity was used. The identities for the mature sequences of all six miRNAs are 100% and the identity of hairpin sequences were 94% for JL-2 and JL-6. JL-1, JL-2, JL-4, JL-5, and JL-6 were located in intergenic regions and JL-3 was located in coding sequence, which is consistent in both assemblies*.

### Expression Profile of the Parasite miRNAs

**FIGURE 2:**
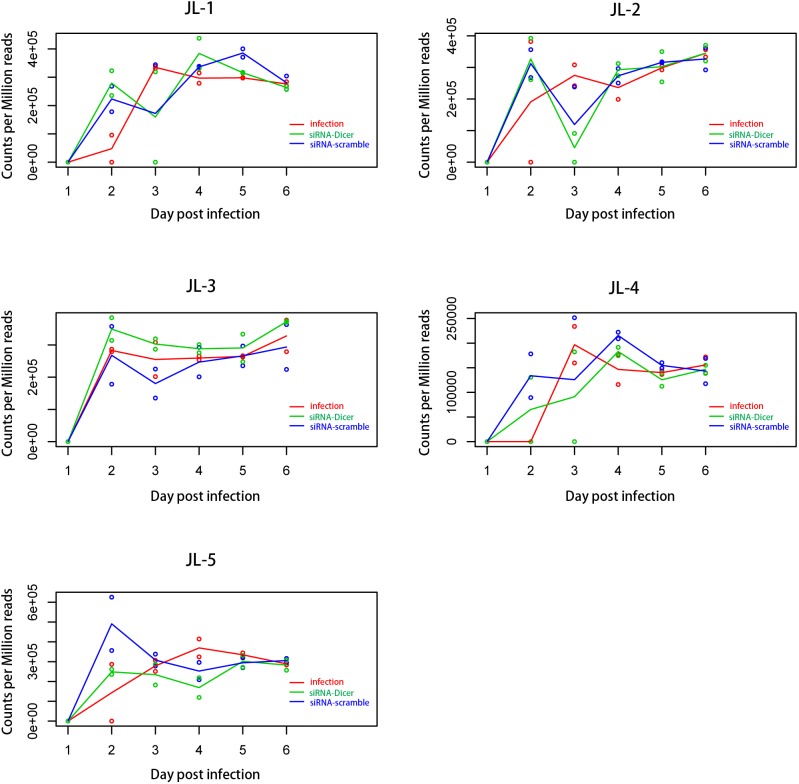
Expression patterns of parasite miRNAs in three treatment groups. JL-1, JL-2, JL-3, JL-4, and Jl-5 showed significantly different expression patterns among the infection group, siRNA-Dicer group and siRNA-scramble group. The expression patterns of JL-3 and JL-5 were significantly different between the siRNA-Dicer and siRNA-scramble groups. The expression patterns of all five miRNAs were significantly different between the infection and siRNA-Dicer groups, as well as between the infection and siRNA-scramble groups, which suggests the random foreign small RNAs had substantial impacts on the expression of parasite miRNAs.

The expression of JL-6 was not detected at any time point. JL-1, JL-2, JL-3, JL-4, and JL-5 were detected as early as 2 dpi. The expression patterns of JL-1, JL-2, JL-3, JL-4, and JL-5 were significantly different among the infection group, siRNA-Dicer group and siRNA-scramble group (*P* < 0.05). Specifically, JL-3 and JL-5 differed significantly between the siRNA-Dicer and siRNA-scramble groups. JL-1, JL-2, JL-3, JL-4, and JL-5 showed significantly expression levels between the infection and siRNA-Dicer groups, as well as between the infection and siRNA-scramble groups (**Figure 2** and **Supplementary File S1**).

### Candidate Target Genes of Parasite miRNAs

**FIGURE 3:**
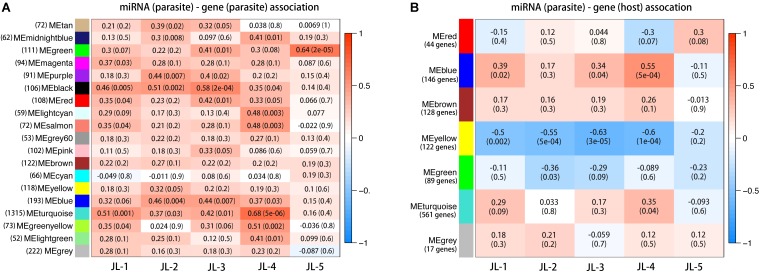
MicroRNA and target gene associations. As it is unclear whether parasite miRNAs target host genes, the candidate genes of both host and parasite were screened. Parasite miRNAs potentially target 918 honey bee genes **(B)** and 2534 parasite genes **(A)**. Each row corresponds to a gene cluster and each column corresponds to a miRNA. Each cell contains the corresponding correlation and *P*-value. The table is color coded by correlation to the color legend on the right side.

It remains unclear whether parasite miRNAs regulate the mRNAs of honey bee or *N. ceranae*. Therefore, candidate target genes of honey bee and *N. ceranae* were both screened. Using a sequence-base method, 1545 honey bee genes were potentially regulated by the five parasite miRNAs, which were clustered into 6 co-expression groups (**Supplementary File S2**). Four (918 genes) out of six co-expression groups were significantly associated, with the expression of at least one miRNA (**Figure 3** and **Supplementary File S3**). Eighteen co-expression groups were clustered from 3219 *N. ceranae* genes. Fourteen groups (2534 genes) were significantly associated with at least one miRNA (**Figure 3**). The distribution of positive and negative associations between parasite miRNAs and target mRNAs was significantly different between host and parasite (*P* < 0.001, Pearson’s Chi-Square test). The parasite miRNAs tended to be positively associated with parasite gene and negatively associated with host genes. Furthermore, the distribution of gene function categories (metabolism, genetic information processing, environmental information processing, cellular process, organismal systems, and human disease) between honey bee genes targeted by parasite versus host miRNAs was also significantly different (*P* < 0.001, Pearson’s Chi-Square test).

**FIGURE 4:**
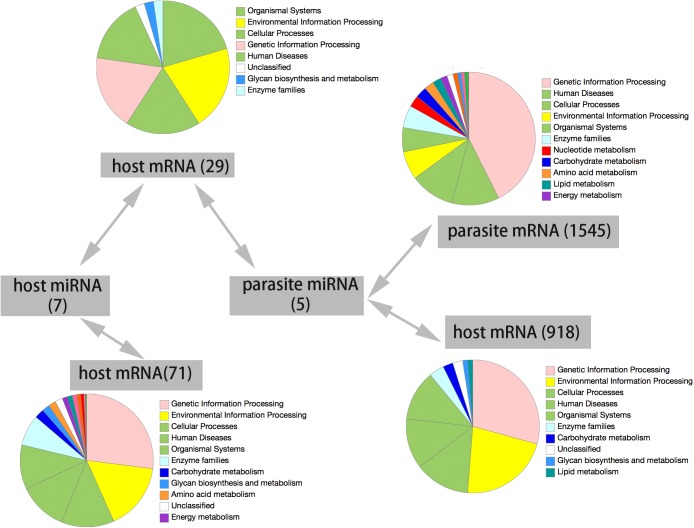
Target genes of miRNAs. Five parasite miRNA could target 1545 parasite gene and 918 host genes. Seven host miRNA could target 71 host genes. There are twenty host genes, which could be targeted by the miRNAs of both host and parasite. The functional category of the genes in each cluster and the corresponding color were shown at the right side according to the KEGG pathway database.

For the parasite, over 78% of total genes were significantly associated with parasitic miRNAs with gene functions including energy metabolism, carbohydrate and lipid metabolism, nucleotide and amino acid metabolism, genetic information processing, and cell reproduction transition (**Figure 4**). Ion binding (GO: 0005509, molecular function), sarcoplasm (GO: 0016528, cellular component) and tissue development (GO: 0009888, biological process) were significantly enriched in host miRNAs regulated genes. In contrast, transcription factor activity (GO: 0000989, molecular function), nucleus (GO: 0005634, cellular component), and cell maintenance (GO: 0019827, biological process) were significantly enriched in parasite regulated host genes. Particularly, the parasite miRNA may even target host RNAi pathway, including RISC-loading complex (GO: 0070578), RNAi effector complex (GO: 0031332), siRNA binding (GO: 0035197), miRNA binding (GO: 0035198), gene silencing (GO: 0016458). There are 29 host genes that were potentially targeted by both host and parasite miRNAs. Enriched functional groups included transport (GO: 0006886), apoptosis (GO: 2001244), and innate immune response (GO: 0002520) (**Figure 4**).

## Discussion

Host–parasite interactions are a reciprocal adaptation process in which parasites need to balance their transmission and virulence (Grosberg and Hart, 2000; Alizon et al., 2009). For an intracellular parasite, all the resources required for proliferation are taken from the host. It is then not surprising that *N. ceranae* modulates honey bee metabolism, immune responses, and the stability of cells for at least one proliferation cycle (Antunez et al., 2009; Mayack and Naug, 2009; Holt et al., 2013; Martín-Hernández et al., 2017). So far, at least two virulence factors have been described, immune suppression and apoptosis inhibition, both of which were implicated in honey bee population that tolerates *N. ceranae* infection (Huang et al., 2012; Kurze et al., 2015). Specific mechanisms responsible for immune and apoptosis suppression remain unclear.

Microsporidia comprise a large group of unicellular parasites that diverged early in the lineage leading to fungi (Capella-Gutiérrez et al., 2012). Microsporidia and a few fungal species are known to have a functional RNAi pathway, suggesting that this trait is ancestral to both lineages (Dang et al., 2011). To date, over 20 microsporidia species have been sequenced and over half have lost the *Dicer* and *Argonaute* orthologs, including the most well studied *Encephalitozoon* and *Nematocida* species. It is challenging to study the RNAi pathway for microsporida, since RNAi mutants are not available (Reinke and Troemel, 2016). Using siRNA or double stranded RNA (dsRNA) is also challenging, because when RNAi gene expression is too low, the RNAi pathway efficiency is by definition compromised. We previously found that honey bee miRNAs respond to *N. ceranae* infection and those miRNAs targeted metabolism (Huang et al., 2015). We also found feeding *N. ceranae* infected honey bee with siRNA targeting *N. ceranae* gene *Dicer*, significantly reduced spore proliferation (Huang et al., 2016). In this study, we found that *N. ceranae* significantly regulated the expression profiles of both host and parasite miRNAs. Host miRNAs regulated by *N. ceranae* infection were not regulated when parasite siRNA-Dicer was present. As *N. ceranae* is the only microsporidian parasite with identified miRNAs, currently, it is crucial to understand genes targeted by parasite miRNA. We were surprised to find that 78% of *N. ceranae* protein-coding genes were potentially regulated by five parasitic miRNA, suggesting that these miRNAs globally influence the parasitic transcriptome. We also found that 918 honey bee genes were potentially targeted by parasitic miRNA, out of which 29 genes can be targeted by both honey bee and *N. ceranae* miRNAs. If *N. ceranae* can secrete miRNA into the host cell cytoplasm, then *N. ceranae* can hijack the honey bee RNAi pathway to suppress honey bee genes (Buck et al., 2014). Genes involved in metabolism appear to be the main targets of parasite miRNAs compared with honey bee miRNAs.

Honey bee genes involved in apoptosis and innate immune response are regulated by the miRNAs of both host and parasite. Apoptosis has been reported as an important defense mechanism toward *N. ceranae* and other microsporidial parasites (del Aguila et al., 2006; Higes et al., 2013; He et al., 2015; Doublet et al., 2017; Martín-Hernández et al., 2017). However, the mechanism of apoptosis regulation is unclear. In our study, the gene *Broad-Complex* (*Br-c, LOC552255*) appears to be targeted by parasite miRNAs. *Br-c* is an important ecdysone gene which is involved in regulating developmental processes and programmed cell death (Tzolovsky et al., 1999; Cakouros et al., 2002). The gene ras-related protein *Rac1* (LOC551554), is involved in transmitting signals within cells, which is important regulator of cell proliferation (McCormick, 1995). Cells with suppressed *Rac* have reduced growth rate and undergo apoptosis. Cell with activation of *Rac* promoted cell growth (Prober and Edgar, 2000). Given *N. ceranae* secreted miRNAs into the cytoplasm, the parasitic miRNA can directly regulate honey bee immune responses and resource transfer. miRNAs of the fungal parasite *Botrytis cinerea* have also been reported to suppress host immune responses (Weiberg et al., 2013), and it will be interesting to compare uses of host and parasite gene regulation pathways in these two systems.

## Ethics Statement

The apiaries for bee sample collection are the property of the USDA-ARS Bee Research Laboratory, Beltsville, MD, United States. No specific permits were required for the described studies. Studies involved the European honey bee (*Apis mellifera*), which is neither an endangered nor a protected species.

## Author Contributions

QH designed the study, carried out the molecular lab work, analyzed the data, and wrote the manuscript. JE designed the study, analyzed the data, and wrote the manuscript. All authors gave final approval for publication.

## Conflict of Interest Statement

The authors declare that the research was conducted in the absence of any commercial or financial relationships that could be construed as a potential conflict of interest.

## Supplementary Material

FILE S1 *Nosema ceranae* and honey bee microRNA (miRNA) expression data.

FILE S2 Target prediction and expression of miRNAs.

FILE S3 *N. ceranae* and honey bee miRNA–mRNA co-expression data.
